# A cohort study of antihypertensive treatments and risk of renal cell cancer

**DOI:** 10.1038/sj.bjc.6602490

**Published:** 2005-04-05

**Authors:** J P Fryzek, A H Poulsen, S P Johnsen, J K McLaughlin, H T Sørensen, S Friis

**Affiliations:** 1 International Epidemiology Institute, Rockville, MD, USA; 2 Department of Medicine, Vanderbilt University Medical Center, Vanderbilt-Ingram Comprehensive Cancer Center, Nashville, TN, USA; 3 Institute of Cancer Epidemiology, Danish Cancer Society, Copenhagen, Denmark; 4 Department of Clinical Epidemiology, Aarhus University Hospital, Aarhus, Denmark; 5 Department of Cardiology, Aaborg Hospital, Aalborg, Denmark

**Keywords:** antihypertensives, diuretics, renal cell cancer incidence, risk, epidemiology, cohort study

## Abstract

We studied 335 682 county residents, of whom 113 298 had been prescribed antihypertensive treatment (AHT), in the period 1989–2002 in North Jutland County, Denmark to examine the relation between different AHTs and the risk of renal cell carcinoma (RCC). An internal comparison was performed among the different classes of AHT users with users of beta blockers as the reference, in order to address potential confounding and bias. The average follow-up was 10 years (range 0–13). Use of any AHT was associated with RCC (relative rate (RR)=1.6, 95% confidence interval (CI) 1.3–1.9) compared with nonusers in the general population. Specific classes of AHTs were nonsignificantly associated with RCC, but compared with users of beta blockers, the numbers observed were close to expectation. Analyses by duration of follow-up and number of prescriptions revealed no clear trends for any antihypertensive agent and after 5-years of follow-up, the RRs for all classes of AHT decreased. The elevated RRs for RCC among users of AHTs compared with the general population are unlikely to be causal, but rather reflect confounding due to failure to control for pre-existing hypertension, and protopathic bias, due to the presence of hypertension as an early sign of kidney disease.

Hypertension is estimated to contribute to one in eight deaths worldwide (WHO, 2002) and up to 60% of affected individuals in the US are on antihypertensive treatment (AHT) (Chobanian *et al*, 2003). Concerns have been raised that certain AHT may increase the risk of renal cell carcinoma (RCC) whether by being directly carcinogenic, by eliciting or accelerating other carcinogens, or by impairing defence mechanisms (Yu *et al*, 1986; McLaughlin *et al*, 1995; Grossman *et al*, 2001).

Past studies have suggested an association between diuretics and RCC, but other AHTs were not studied as extensively. Though we have reported on use of calcium channel blockers and ACE inhibitors in relation to cancer in general in North Jutland County, Denmark, we did not look in any detail at RCC risk in relation to the wide variety of specific AHTs (Olsen *et al*, 1997; Sorensen *et al*, 2000; Friis *et al*, 2001); these earlier studies had relatively short follow-up periods, ending in 1993, 1995 and 1996, respectively. The current study updates the follow-up period through 2002 and examines the relation between the all five major classes of AHTs (Capella, 1993), that is, diuretics, beta blockers, calcium antagonists, angiotensin converting enzymes (ACE) inhibitors and angiotensin II antagonists, as well as specific classes of diuretics, in relation to RCC.

## MATERIALS AND METHODS

### Study population

The study was conducted among 335 682 residents of North Jutland County, Denmark, with no prior diagnosis of any type of cancer, between 30 and 85 years old, and resident in the county on 1 January 1989. Age was restricted to those between 30 and 85 years old because of the limited number of people outside these ages who were first time users of AHT. Of these, 113 298 had been prescribed an AHT between the years 1989 and 2002. These data sources have been described in previous studies in this population (Olsen *et al*, 1997; Sorensen *et al*, 2000; Friis *et al*, 2001)

### Exposure group

Prescriptions of AHT medications were ascertained from the Pharmacoepidemiological Prescription Database for individuals in the study population between the years 1989 and 2002. Along with type of drug prescribed according to the Anatomical Therapeutical Chemical (ATC) classification system (Capella, 1993), date of dispensing at the pharmacy and the patient's civil registry number were abstracted from this database. In an attempt to address the problem of noncompliance, we required two or more AHT prescriptions recorded in the prescription database during the study period for a person to be classified as exposed. To ensure correct identification of exposure status for those who might have started AHT therapy prior to 1989, 1989 data were used only to assess exposure status, and follow-up was initiated in 1990.

AHT medications were recorded in the pharmacy database by their ATC code, managed and developed by the World Health Organization (WHO, 2004). In this classification system, the drugs are divided into different groups according to the organ or system on which they act and their chemical, pharmacological and therapeutic properties. For this study, we identified all prescriptions filled for AHT medications, including diuretics (ATC group=C03), beta blockers (ATC group=C07), calcium channel blockers (ATC group=C02D, C08C, C08D (minus C08DA51)), ACE inhibitors (ATC group=C02E, C02L, C09A, C09BA) and angiotensin II antagonists (ATC group=C09C, C09D). In addition, specific types of diuretics were examined. These included thiazides (ATC group=C03A); sulphonamides, mercurial diuretics, xanthine derivatives and other low-ceiling diuretics except thiazides (ATC group=C03B); loop (or high-ceiling diuretics) (ATC group=C03C); potassium-sparing agents (ATC group=C03D); and diuretics and potassium-sparing agents in combination (ATC group=C03E).

### Renal cell cancer (RCC)

Cases of RCC had either an ICD7 code of 180.0 (kidney cancer, not otherwise specified) or 180.3 (renal cell cancer). ‘Kidney cancers, not otherwise specified’, were regarded as situated in the renal parenchyma as a previous validation study indicated that most of these tumours are RCC (Mellemgaard *et al*, 1993). We excluded cancers of the renal pelvis and ureter (ICD7=180.1, 180.2) as their aetiology resembles that of bladder cancer (McLaughlin and Lipworth, 2000).

### Follow-up

Follow-up began on the date of the second prescription for AHT medication or 1 January 1990, whichever was later. Follow-up ended at diagnosis of RCC, age 85, death, emigration from the county, or 31 December 2002, whichever was earliest. The person-time of the study subjects was distributed according to use of AHT medications in exposed time (⩾2 prescriptions for AHT) (AHT users) and unexposed time (one or no prescriptions for any AHT) (nonusers).

### Statistical analysis

Simple descriptive statistics, including frequencies, percentages and means, were calculated to describe the study cohort. Log-linear Poisson regression analysis with the logarithm of person-years at risk as an offset variable was used to estimate the rate ratios associated with use of each type of AHT medication. All models were adjusted for calendar period (1990–1994 or 1995–2002), gender and age group (30–49, 50–59, 60–69 or 70–85). Subjects could change between categories of covariates and specific types of AHT over time, except for the analyses of ‘exclusive users’ where subjects did not change between categories of specific types of AHT use. In order to control for potential unmeasured confounding or bias in risk estimates for users of AHT medication based on comparisons with the general population, we also performed a direct comparison between users of a specific AHT and beta blockers. Beta-blockers' pharmacodynamic ‘target organs’ are outside the kidney (namely heart and vessels). These direct comparisons were restricted to ‘exclusive users’ of the various AHT medications, including the reference category of beta blockers. Beta blockers were chosen as the reference group because there is no consistent evidence that use of these compounds is associated with increased risk of cancer (Grossman *et al*, 2001). Since many of these drugs could be used in combination, we also performed analyses for ‘exclusive users’ of specific AHT medications.

Further stratified analyses on the whole study population comparing AHT users and the general population for duration of follow-up (1, 1–4 and 5 or more years) and number of prescriptions (2–4, 5–9, 10+ prescriptions) were also performed. Within each categorical level all variables were treated as time independent. The statistical analysis was performed in SAS 8.02.

## RESULTS

For the 335 682 people in the study population, the average follow-up was 10 years (range=0–13 years). The 113 298 people prescribed two or more AHT prescriptions (exposed cohort) accrued a total of 706 428 person-years of follow-up, yielding an average follow-up of 6 years (range=0–13 years). Other characteristics of AHT users are shown in Table 1. More women (58%) than men (42%) were prescribed AHT and, as expected, most of the prescriptions were for people over age 50 (77%) with the average age of entry into the AHT user cohort (the date of the second AHT prescription) of 62 years (range=30–85 years). More than half of the cohort (53%) had received 20 or more prescriptions for AHT. Diuretics were the most common type of AHT medication prescribed (25%), most of which were thiazides. Beta blockers and calcium antagonist were prescribed for at least 10% of the population of AHT users, while fewer prescriptions were filled for ACE inhibitors and angiotensin II antagonists.

After controlling for age, gender and calendar period, users of any type of AHT were 1.6 times (95% CI=1.3–1.9) more likely to have RCC compared with individuals in the general population who did not use AHTs (Table 2). Use of ACE inhibitors (relative rate (RR)=1.9, 95% CI=1.4–2.5), beta blockers (RR=1.7, 95% CI=1.3–2.2), calcium antagonist (RR=1.4, 95% CI=1.0–1.8) and diuretics (RR=1.6, 95% CI=1.2–2.0) were all significantly associated with RCC. Furthermore, the risk for RCC among each specific class of diuretics was elevated, with a significant association for loop diuretics (RR=2.0, 95% CI=1.5–2.7), and potassium-sparing agents (RR=2.2, 95% CI=1.3–3.8).

Next, we examined the relation between exclusive use of a specific class of AHT and RCC risk. Most of the relative risks were attenuated but slight to moderately elevated risks were still present. However, there were no significant differences in RCC risk among different classes of drugs when exclusive users of specific AHT medications were compared with beta blocker users, with risks of 1.2 (95% CI=0.4–3.3) for ACE inhibitors, 1.0 (95% CI=0.1–7.5) for angiotensin II antagonist, 0.9 (95% CI=0.4–1.9) for calcium antagonist, and 0.9 (95% CI=0.5–1.6) for diuretics.

Table 3 presents the association between the various classes of AHT and RCC by duration of follow-up using the general population as the comparison group. Overall, the relation between AHT use and RCC decreased over time (RR for <1 year=2.0, 95% CI=1.4–2.9; RR for 1–4 years=1.6, 95% CI=1.2–2.0; RR for 5 and more years=1.4, 95% CI=1.0–1.8). A similar pattern of decreasing risk with increasing duration of follow-up was found for ACE inhibitors, angiotensin II antagonist, beta blockers and calcium antagonists. Overall, for the various classes of diuretics, no trends of increasing or decreasing risks were apparent over time. None of the specific types of AHT drugs was significantly associated with RCC after 5 or more years of follow-up.

Overall, no increasing or decreasing trend in RCC risk with increasing number of prescriptions for AHT medications was apparent (Table 4). Similar patterns were seen for the specific classes of AHT and diuretics in particular. RRs were remarkably similar for individuals with 10 or more prescriptions for virtually all classes of AHT medications with point estimates ranging between 1.4 and 1.8, and for the various types of diuretics, with point estimates ranging from 1.0 to 1.7.

## DISCUSSION

Although, there was an increased risk for RCC among users of AHT medications compared with nonusers in the general population, risk for exclusive use of any class of AHT medication was not significantly elevated compared with either the general population or with users of beta blockers. In fact, in comparison with beta blockers, the risk estimates were close to unity for all major groups of AHT medications. Neither duration of follow-up nor number of prescriptions was associated with RCC for any of the classes of AHT medications.

It is known that early-stage prediagnostic renal tumours may themselves lead to increases in blood pressure and the observed association between AHT medication and RCC is likely to reflect to some extent this type of protopathic bias. Our data lend some support for this view. After 5-years of follow-up, the risk ratios for all classes of AHT medications decreased, indicating that the onset of hypertension as an early sign of kidney cancers rather than the treatment *per se* is related to RCC.

Numerous observational studies have suggested that elevated risk ratios, may, in fact, reflect a relation between pre-existing high blood pressure and RCC. Thus, failure to adjust for confounding by pre-existing hypertension in our study may have generated inflated risk estimates. When adjustment for high blood pressure is performed, it appears to eliminate the excess risk associated with diuretic use (McLaughlin *et al*, 1995). We examined the specific classes of diuretics and found similar risks across all groups. As the five classes of diuretics have different modes of action, it is reasonable to assume that if these medications were related to RCC, risk would differ across the specific classes of diuretics. This was not the case in our study, as the risk for RCC was similarly elevated for all classes of diuretics. When compared with beta blockers, the risk for RCC among each group of diuretics except potassium-sparing agents was near unity and no trend in risk was apparent with increasing duration of follow-up or number of prescriptions. Only one case of RCC was diagnosed among users of potassium-sparing agents. Based on past studies in this population and others, it is unlikely that calcium antagonists or beta blockers, or ACE inhibitors are associated with cancer (McLaughlin *et al*, 1995; Olsen *et al*, 1997; McLaughlin and Lipworth, 2000; Sorensen *et al*, 2000; Friis *et al*, 2001; Grossman *et al*, 2001). To our knowledge this is the first study to examine the relation between angiotensin II antagonists and RCC.

The strengths of the study include the large size with over 3 million person-years of follow up and 471 cases of RCC resulting in an improved statistical precision compared with earlier studies, and complete follow-up of the cohort through use of the unique CPR number with computerised linkage to nationwide Danish registers. A further strength is that beta-blockers are available in Denmark only by prescription, and we have identified practically all users in the study population. The limitations include the fact that the prescription database was only established in 1989 so earlier drug history is unknown. Information on well known or suspected risk factors for RCC, such as cigarette smoking, obesity and possibly hypertension was not available. Several large-scale population-based case–control and cohort studies which adjusted for use of diuretics and other AHT have reported elevated relative risks for RCC associated with hypertension, independent of use of AHT (McLaughlin *et al*, 1995; Yuan *et al*, 1998; Chow *et al*, 2000; McLaughlin and Lipworth, 2000). In contrast, adjustment for high blood pressure appears to eliminate any excess risk for RCC associated with AHT use (McLaughlin *et al*, 1995; Yuan *et al*, 1998; Shapiro *et al*, 1999; McLaughlin and Lipworth, 2000). In an attempt to control for potential confounding variables, we compared beta blockers and other medications and found that all of the risk estimates were close to expectation.

Diabetes has been suggested as a risk factor for RCC, but the evidence is not consistent (Wideroff *et al*, 1997; Coughlin *et al*, 2004). Exclusion of diabetics did not materially change the risk estimates (data not shown), suggesting that diabetes does not play a major role in the aetiology of RCC in this cohort.

In summary, none of the AHTs studied was consistently associated with risk of RCC, and analyses by duration of follow-up and number of prescriptions indicated that it is unlikely that any of these medications plays an important role in the aetiology of RCC. Elevated RRs for RCC among of AHT users compared with the general population are unlikely to be causal, but rather reflect confounding, from failure to control for pre-existing hypertension, which appears to be an independent risk factor for RCC, from hypertension as an early sign of kidney cancer and possibly from increased medical surveillance.

## Figures and Tables

**Table 1:** Characteristics of users of antihypertensive medications, North Jutland, Denmark, 1989–2002

**Characteristic**	**Antihypertensive medication users^a^**	
	**Number**	**Percent**
*Total*	113 298	(100)
Men	47 373	(42)
Women	65 925	(58)

*Age at entry* b		
<50 years	25 900	(23)
50–69 years	49 768	(44)
⩾70 years	37 630	(33)

Mean age at entry (range)^b^	62	(30–85)

*Year of entry* b		
1990–1992	53 139	(47)
1993–1995	20 786	(18)
1996–1998	16 718	(15)
1999–2002	22 655	(20)

*Number of prescriptions for any antihypertensive medication*		
2–5	21 294	(19)
6–10	13 688	(12)
11–20	18 821	(17)
⩾20	59 495	(53)

*Type of antihypertensive medication* c		
Any antihypertensive	113 298	(34)
ACE inhibitor	29 486	(9)
Angiotensin II antagonist	13 755	(4)
Beta blocker	42 866	(13)
Calcium antagonist	34 816	(10)
Diuretic	83 060	(25)
Low-ceiling diuretics, thiazides	45 714	(14)
Sulphonamides and other low-ceiling diuretics	4976	(1)
Loop (high-ceiling) diuretics	36 197	(11)
Potassium-sparing agents	7894	(2)
Diuretics and potassium-sparing agents in combination	15 582	(5)

a Two or more prescriptions for antihypertensive medications.

b Date of second prescription.

c Since cohort members may use more than one antihypertensive medication, the total number exceeds the total number of people in the cohort.

**Table 2:** Relative rates (RR) and 95% confidence intervals (CI) for the relation between antihypertensive medication use and renal cell cancer (RCC) for individuals who ever used a specific class of antihypertensive medication and for those who exclusively used a specific class of antihypertensive medication compared to the general population or to people who use beta blockers in North Jutland County, 1989–2002

	**Ever used antihypertensive medication**				**Exclusive use of specific class of antihypertensive medication**					
		**General population**				**General population**			**Beta blockers**	
	**RCC cases**	**Person- years**	**RR^a^**	**(95% CI)**	**RCC cases**	**Person- years**	**RR^a^**	**(95% CI)**	**RR^a^**	**(95% CI)**
Any antihypertensive	191	706 428	1.6	(1.3–1.9)						
ACE inhibitor	52	147 096	1.9	(1.4–2.5)	5	18 545	1.8	(0.8–4.5)	1.2	(0.4–3.3)
Angiotensin II antagonist	9	43 456	1.1	(0.6–2.2)	1	4958	1.4	(0.2–10.2)	1.0	(0.1–7.5)
Beta blocker	72	245 971	1.7	(1.3–2.2)	15	73 179	1.6	(0.9–2.7)	1.0	(Referent)
Calcium antagonist	59	195 715	1.4	(1.0–1.8)	9	34 915	1.3	(0.6–2.5)	0.9	(0.4–1.9)
Diuretic	138	489 470	1.6	(1.2–2.0)	55	236 455	1.4	(1.0–1.9)	0.9	(0.5–1.6)
Thiazides	62	248 890	1.3	(0.9–1.7)	14	77 421	1.0	(0.6–1.7)	0.7	(0.3–1.5)
Sulphonamides and other low-ceiling diuretics	9	35 407	1.4	(0.7–2.7)	3	11 482	1.6	(0.5–5.0)	1.1	(0.3–3.7)
Loop (high-ceiling) diuretics	70	168 311	2.0	(1.5–2.7)	13	41 785	1.6	(0.9–2.8)	1.1	(0.5–2.4)
Potassium-sparing agents	15	30 550	2.2	(1.3–3.8)	1	1164	5.0	(0.7–36.0)	3.3	(0.4–25.1)
Diuretics and potassium-sparing agents in combination	25	111 436	1.3	(0.8–1.9)	4	40 316	0.6	(0.2–1.6)	0.4	(0.1–1.2)

a Adjusted for age, gender and calendar period.

**Table 3:** Relative rates (RR) and 95% confidence intervals (CI) for the relation between antihypertensive medication use and renal cell cancer (RCC) by years of follow-up in North Jutland County, 1989–2002

**Years of follow-up**	**<1 year**				**1–4 years**				**5+ years**			
	**Number RCC**	**Person- years**	**RR^a^**	**95% CI**	**Number RCC**	**Person- years**	**RR^a^**	**95% CI**	**Number RCC**	**Person- years**	**RR^a^**	**95% CI**
Any hypertensive	33	95 484	2.0	(1.4–2.9)	89	323 680	1.6	(1.2–2.0)	69	287 264	1.4	(1.0–1.8)
ACE inhibitor	12	26 154	2.3	(1.3–4.2)	30	71 859	2.2	(1.5–3.2)	10	49 084	1.1	(0.6–2.1)
Angiotensin II antagonist	4	12 072	1.9	(0.7–5.0)	5	27 022	1.0	(0.4–2.5)	0	4362	0.0	—
Beta blocker	14	37 211	2.2	(1.3–3.8)	34	114 335	1.7	(1.2–2.5)	24	94 425	1.4	(0.9–2.1)
Calcium antagonist	9	30 749	1.4	(0.7–2.7)	31	96 677	1.5	(1.0–2.2)	19	68 288	1.2	(0.7–2.0)
Diuretic	22	71 157	1.7	(1.1–2.6)	70	227 812	1.7	(1.3–2.2)	46	190 501	1.4	(1.0–1.9)
Thiazides	7	40 613	0.9	(0.4–1.9)	36	120 761	1.5	(1.1–2.2)	19	87 516	1.1	(0.7–1.8)
Sulphonamides and other low-ceiling diuretics	1	4257	1.3	(0.2–9.2)	3	15 006	1.1	(0.3–3.4)	5	16 144	1.7	(0.7–4.1)
Loop (high-ceiling) diuretics	17	30 512	2.5	(1.5–4.1)	37	84 227	2.1	(1.5–3.0)	16	53 572	1.6	(0.9–2.7)
Potassium-sparing agents	9	6338	6.0	(3.1–11.8)	5	14 607	1.5	(0.6–3.7)	1	9606	0.5	(0.1–3.6)
Diuretics and potassium-sparing agents in combination	5	13 457	2.1	(0.9–5.1)	12	48 587	1.4	(0.8–2.5)	8	49 393	0.9	(0.4–1.8)

a All analyses adjusted for age, gender and calendar period.

**Table 4:** Relative rates (RR) and 95% confidence intervals (CI) for the relation between antihypertensive medication use and renal cell cancer (RCC) by number of prescriptions in North Jutland County, 1989–2002

**Years of follow-up**	**2–4 prescriptions**				**5–9 prescriptions**				**10+ prescriptions**			
	**Number RCC**	**Person- years**	**RR^a^**	**95% CI**	**Number RCC**	**Person- years**	**RR^a^**	**95% CI**	**Number RCC**	**Person- years**	**RR^a^**	**95% CI**
Any hypertensive	34	144 503	1.6	(1.1–2.4)	28	121 168	1.4	(0.9–2.1)	129	440 757	1.6	(1.3–2.0)
ACE inhibitor	9	33 171	1.5	(0.8–2.9)	14	31 214	2.4	(1.4–4.1)	29	82 712	1.8	(1.2–2.7)
Angiotensin II antagonist	4	10 604	2.1	(0.8–5.7)	2	11 779	0.9	(0.2–3.8)	3	21 074	0.8	(0.2–2.4)
Beta blocker	25	66 398	2.4	(1.6–3.7)	10	55 998	1.0	(0.5–1.9)	37	123 576	1.6	(1.1–2.3)
Calcium antagonist	10	41 033	1.2	(0.6–2.3)	11	39 441	1.3	(0.7–2.4)	38	115 241	1.4	(1.0–2.0)
Diuretic	28	135 964	1.3	(0.9–1.9)	30	108 244	1.5	(1.0–2.3)	80	245 261	1.7	(1.3–2.2)
Low-ceiling diuretics, thiazides	14	86 346	0.9	(0.5–1.6)	17	65 314	1.3	(0.8–2.2)	31	97 230	1.5	(1.0–2.2)
Sulphonamides and other low-ceiling diuretics	5	12 202	2.5	(1.0–6.1)	1	8788	0.6	(0.1–4.4)	3	14 418	1.0	(0.3–3.3)
Loop (high-ceiling) diuretics	18	52 196	1.9	(1.1–3.0)	23	36 850	3.0	(1.9–4.6)	29	79 265	1.7	(1.1–2.5)
Potassium-sparing agents	8	9068	4.0	(2.0–8.1)	2	7376	1.2	(0.3–4.8)	5	14 106	1.6	(0.7–4.0)
Diuretics and potassium-sparing agents in combination	9	34 157	1.6	(0.8–3.1)	6	24 606	1.3	(0.6–3.0)	10	52 674	1.0	(0.5–1.9)

a All analyses adjusted for age, gender and calendar period.
